# The early work on the discovery of the function of the thymus, an interview with Jacques Miller

**DOI:** 10.1038/s41418-019-0462-y

**Published:** 2019-12-05

**Authors:** Jacques Miller

**Affiliations:** The Walter and Eliza Hall Institute, 1G Royal Parade Parkville, Parkville, VIC Australia

## Abstract

This interview is part of a series of articles to mark the 25th anniversary of *Cell Death and Differentiation*.

The thymus was the last major organ to have its function discovered in 1961. Thymus-derived cells (now known as T cells) were shown to mediate immune responses. The presence of T cells in human cancers showed that they had the potential to destroy tumor cells. More recently, a large family of T-cell subsets with different functions have been identified. Here, *Cell Death and Differentiation* asks Jacques Miller about his early work on thymus and T cells.

**Fig. 1:**
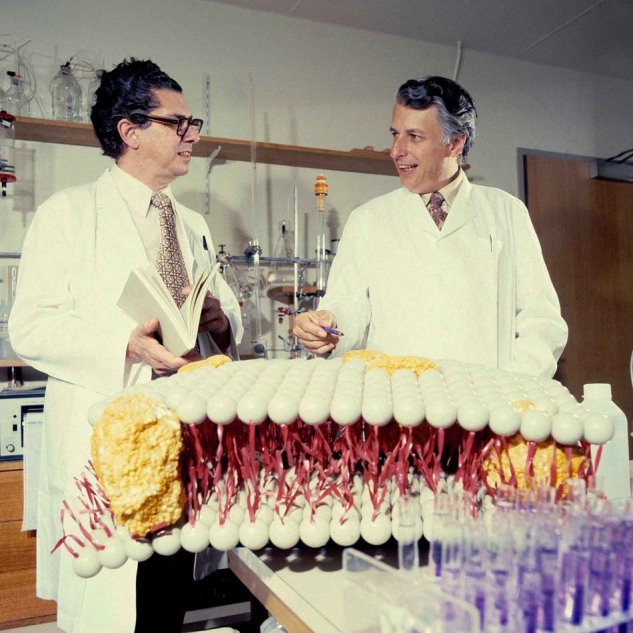
Jacques (left), Gus Nossal (right) in 1967. In 1966, Gus Nossal succeeded Sir MacFarlane Burnet as director of the Walter and Eliza Hall Institute of Medical Research (WEHI) in Melbourne, Australia. He invited Jacques Miller (then at the Institute of Cancer Research in London) to head a new Laboratory at WEHI in Melbourne, Australia.

CDD: Could you please describe your personal background, who were your parents, where were you born and what happened in your early years?

My father and mother were both born in Paris in 1896. During the first World War (1914–1918), my father, Maurice Meunier, who spoke English fluently, acted as interpreter for the British troops who came to France. In 1919, he married and left with his new wife for China having found a job in a French bank in Peking (now known as Beijing). He spent some 22 years in China and Japan, eventually becoming Manager of the Franco-Chinese Bank in Shanghai. Besides English, he also spoke Spanish fluently, and learned Mandarin Chinese which he could write, and also Japanese which he wrote and spoke.

In 1930, my mother returned to France by ship for health reasons. Finding that she was pregnant, she decided to have the baby in France and so, having been conceived in China, I was born in France, in Nice, in April 1931. In 1932, she went back to China with her three children, Jacqueline, the eldest, Jeanine her second and me. She was back in France in 1935, both for her health and to allow Jacqueline to receive what she thought would be a good education at a boarding school. Some months later, when we were just about to return to China, Jacqueline was diagnosed with pulmonary tuberculosis. Because of this, the family decided to go to Switzerland which, in those days, was the place where tuberculosis could best be managed. We spent 3 years in Vennes-sur-Lausanne, in a beautiful chalet with an unimpeded view of Mt Blanc, and I do remember my sister Jeanine and I playing together with Jacqueline, even when she was coughing blood stained sputum.

In March 1939, my father joined us on a long service leave, but when World War II broke out 6 months later, he was recalled to Shanghai. Believing that Switzerland would be invaded, he decided that the whole family should return to China. We left Lausanne by car very quickly, crossing Northern Italy on our way to Trieste, and there managed to get the last passenger boat out of Italy.

CDD: Tell us about your time in China and how you ended up in Australia.

We lived in a spacious house about 30  min by car from Shanghai’s central business district. The Franco-Chinese bank where my father worked was located in the French Concession, next to the British Concession. Whenever my mother wanted to go shopping, my father drove us there. I did not like the rampant poverty and obvious disease deformities which plagued many of the Chinese at that time, and I was glad not to have to go to school during my time there.

When France capitulated in 1940, the French Concession was automatically taken over by Vichy officials. My father, who did not accept France’s surrender, rallied to the Gaullists and became active politically. He secretly smuggled young Frenchmen, who wanted to join the British forces, out of the French concession onto British ships leaving for Britain. In 1940, he was actually invited by the British War Office to join the London Headquarters as a link between the French and British Treasury. But in December of that year, only a few years before the discovery of streptomycin, Jacqueline died, aged 17. Because of this, and as these were the months of the blitz in London, my father finally declined the offer from London for the family’s sake. However, it was evident that he had to leave Shanghai, for he was next on the list of Gaullists to be arrested by Vichy officials. He also knew from his knowledge of Japanese, that Japan would enter the war very soon, and that he would be at great risk, as he spoke and wrote their language fluently. Some deal was made with the British authorities in Shanghai: we were given British passports and our surname was translated into English - hence Miller. We left in August 1941, taking the last cargo boat out of Shanghai bound for Batavia (now known as Jakarta). There we boarded a passenger ship and arrived in Sydney on the 25th of September 1941, just less than 3 months before the bombing of Pearl Harbor.

CDD: Tell us about your early time in Australia and your school years.

The Australians in Sydney did not recognize French banking credentials and would not employ my father on an equal footing. Because of this, he founded, together with another Frenchman, the “Free French Delegation”. It took over the activities of the previous consulate, at that time defunct since Australia did not recognize Vichy. He offered his services to the Australian Government, and in fact did translate Japanese documents as requested. He was also active in the war effort for the American forces and he helped with the taking in of supplies to New Caledonia.

Prior to arriving in Australia, Jeanine and I had never been to school. We had teachers at home, wherever we lived. The last one in Shanghai was a 36-year old Viennese with a PhD who had escaped a Nazi prison. Instead of learning much from him, we spent our time having great fun with him. So, we arrived Australia knowing only a very few words of English!

Because my father had been impressed by the knowledge, culture and broad mindedness of the Jesuits in Shanghai, with whom any subject could be discussed, he decided that I should go to a Jesuit college, St Aloysius’ College, and Jeanine went to a convent close by.

At Aloysius I met and frequented a brilliant young Austrian boy, a refugee from Vienna, who was a year ahead of me. His name is Gus Nossal and we became life-long friends. I have followed one year behind in his footsteps first at school, then at the Sydney Medical School, and finally at the Royal Prince Alfred Hospital in Sydney.

CDD: Could you now describe the background in which you got interested in Medicine and Medical research?

I had grown up during World War II and had a great distaste for violence and war. I thought that if such things happened again, when I reached adulthood, I would prefer to be in a position to patch up the wounded. I also had another reason. During Jacqueline’s illness, I had overheard conversations between the doctor and my mother, when he explained to her what tuberculosis was and how little was known about how the body resisted infection. That and the fact that neither Jeanine nor I contracted the disease, even though we had been in close contact with Jacqueline, aroused my curiosity. I thus decided on a Medical career and, if possible, follow this in a research setting.

During my medical studies at Sydney University, I was pleased to interrupt them to do a year’s research as a B.Med.Science student in the laboratory of Professor de Burgh, again following in the footsteps of Gus Nossal. I too was given the task of deciphering how ectromelia virus multiplied in liver cells, but rather than continuing on the line of work that previous B.Med.Science students had performed with normal liver, I thought it more interesting to determine whether the virus might interfere with some crucial biochemical events during liver regeneration after partial hepatectomy. Two papers resulted from this work.

CDD: How did you end up coming to the United Kingdom?

After receiving my medical degree and doing a year as a junior medical resident in Hospital, followed by another year in the Pathology Department of Sydney University, I applied for what was called a Gaggin Research Fellowship, advertised in the Medical Journal of Australia. It was given by the University of Queensland, and offered a return fare to the United Kingdom and a salary for 2 years in a Research Institute of the candidate’s own choosing. I was lucky to get this Fellowship and I applied to many Institutes in England. Most were unable to take me, but one, the Chester Beatty Research Institute, the Institute of Cancer Research, in South Kensington, London, accepted me as a post-doctoral student for the PhD degree of the University of London.

CDD: Did you choose what line of research you might be interested in?

I arrived in the U.K. in 1958, not knowing exactly what I was going to do. Many scientists at the Institute were heavily involved in chemical carcinogenesis, searching for unknown ones and determining their mode of action at the DNA level. This did not interest me much, as I would rather have used the experience I gained in my B.Med.Sci. year to work on some model in which pathogenetic mechanisms had to be investigated. Hence, I felt rather frustrated and did not really want to work in any of those laboratories. I was then told that the Institute had two satellites outside greater London, one being at a place called Pollards Wood, at Chalfont St Giles in Buckinghamshire. There, Dr R.J.C. Harris was working on the development of sarcomas in turkeys caused by the Rous sarcoma virus. I thought that this line of investigation might interest me, and I visited him. Instead of joining his group and working with the Rous virus, he suggested that I might be willing to investigate the pathogenesis of lymphocytic leukemia induced in mice by what was presumed to be a virus that had recently been discovered by Ludwik Gross in the United States. This suited me perfectly.

As a Ph.D. student, Dr Harris was meant to act as my supervisor, although my official supervisor had to be a full Professor of the University of London, which in my case was Professor Sir Alexander Haddow, the director of the Chester Beatty Research Institute. Barely a few months after my arrival, Dr Harris was offered a much better position at the National Institute for Medical Research in Mill Hill near London. I was therefore left without a close supervisor but lucky to inherit his animal space.

Pollards Wood was a large estate that had previously belonged to Bertram Mills, a circus owner. It had a magnificent Tudor-style mansion situated in the middle of beautiful gardens and woods. The rooms had been refurbished to well-equipped laboratories and offices. Scattered throughout the estate were buildings that had previously housed animals such as horses, dogs and elephants. They had also been converted to laboratories or animal quarters. A van from the main Institute in South Kensington came once or twice every week to bring mail and whatever supplies were required. Even though I was given only a small shack and a small amount of space in one of the converted horse stables, it was a delight to work in such pleasant surroundings, away from the crowd, the noise, and the pollution of greater London.

CDD—Why did you research the immune system?

For my PhD degree I worked on virus-induced lymphocytic leukemia, a cancer that arises in the thymus and then spreads. This leukemia could be induced in low-leukemic strain mice by a virus, but only if the virus was injected immediately after birth, not later. I hypothesized that the virus could multiply only in certain cell types present in the developing thymus of neonatal mice. To test this, I removed the thymus surgically (thymectomized) from newborn mice and then injected the virus immediately after thymectomy. At subsequent intervals thereafter, I transplanted a thymus of the same mouse strain anticipating that the transplant would not undergo neoplastic transformation, in contrast to what happened in euthymic neonatally injected mice, thymectomized at 8 weeks of age and then grafted with thymus tissue. What I noticed was totally unexpected. The neonatally thymectomized (NTx) mice became sick some weeks after weaning regardless of whether they had been inoculated with virus. Such illness had never been reported in numerous studies in which adult mice had been thymectomized. This made me conclude that the thymus “at birth may be essential to life”.

CDD—What was known about thymus function when you began your PhD?

The thymus was considered to be a vestigial organ with no known function. It was believed to have become redundant during the course of evolution and to act as a graveyard for dying lymphocytes.

CDD—How did you identify thymus immune function?

I performed autopsies on the NTx mice that had become sick. They had liver lesions suggesting infection by hepatitis virus and a marked diminution of lymphocytes in blood, lymph nodes, and spleen. As lymphocytes from spleen, lymph nodes, blood and lymph, but not from thymus, had just previously been shown by Jim Gowans to be immunologically competent cells, I tested my NTx mice and sham-operated controls for immune function by transplanting foreign skin taken from other mouse strains and from rats. The results were spectacular: the NTx mice failed to reject such skin, whereas the controls did as normal mice would. The NTx mice also failed to produce antibody to many, though not all, antigens. These results were published in 1961 and 1962.

CDD—Could immune function be restored?

Yes, by grafting a thymus taken from the same mouse strain.

CDD—So what happened if a foreign thymus was transplanted?

The immune response of NTx mice grafted with a foreign thymus was restored except to skin grafts derived from the donor strain. This led me to suggest that “when one is inducing a state of immunological tolerance, one is in effect performing a thymectomy, not a complete thymectomy, but a partial, selective or immunological thymectomy”. In other words, thymic lymphocytes developing in the presence of foreign antigens would be deleted, implying that the thymus may be the seat where tolerance to self-components is learned.

CDD—Why does adult thymectomy not cause problems?

I hypothesized that the thymus in adult life might still be essential to enable recovery of lymphoid tissue after this had been damaged, for example following irradiation. This proved to be the case as published in my 1962 Nature paper: the immune system of thymectomized and irradiated mice was unable to recover in contrast to that of irradiated sham-operated controls. Thymectomy of unirradiated adult mice did not cause any immediate defects, although it did produce some degree of lymphopenia several months later.

CDD—So did you show that thymus lymphocytes leave the thymus?

At that time, we had no known genetic markers except those determined by tissue compatibility differences and by a unique mouse strain, “T6”, that had two minute chromosomes visible during metaphase. I grafted T6 NTx mice with thymus from non T6 strains and detected thymus graft derived cells in the lymphoid tissues after immunization with foreign skin grafts.

CDD—How did the Immunological community react to these findings?

My data were not criticised, but my interpretation was questioned. Some argued that perhaps my mice, having been raised in converted horse stables, must have been exposed to so many intercurrent infections that the additional trauma of neonatal or of adult thymectomy followed by irradiation, precipitated immunodeficiency. This prompted me to go to the National Institutes of Health in Bethesda in 1963 to repeat the work in germfree tanks. Germfree mice were neonatally thymectomized or sham operated in the tank and grafted with foreign skin. None became sick and none of the NTx mice rejected the skin (Fig. 2).

**Fig. 2:**
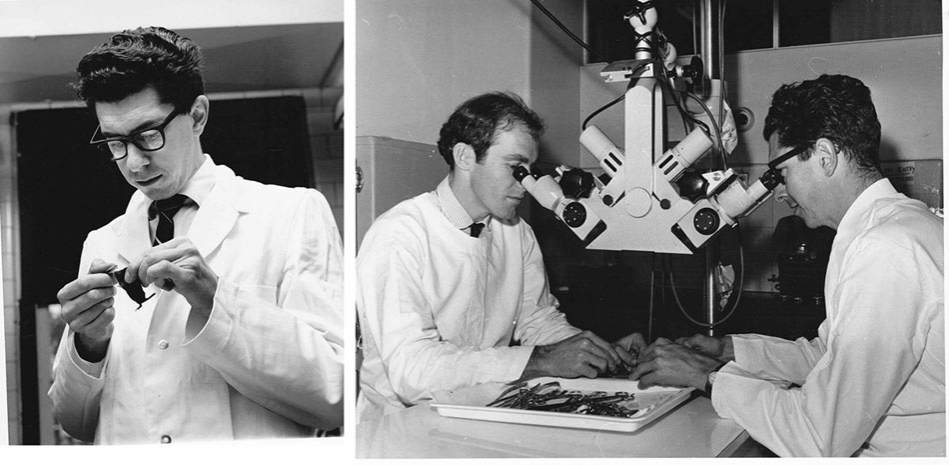
Left, Jacques in 1963. Right, Jacques (right) with Graham Mitchel, performing thoracic duct cannulation in 1968.

CDD—Are all lymphocytes derived from the thymus?

No. My first PhD student, Graham Mitchell, and I clearly showed that thymus derived cells, now called T cells, were not able to produce antibody, but were required to help other lymphocytes derived from bone marrow, now called B cells, to secrete antibody. T and B cells thus cooperated in response to a challenge with many, though not all, antigens.

CDD—Is the T-cell population uniform?

No. The T-cell population has been shown by numerous investigators to consist of many different subsets, each of which plays a distinct role in immune responses. They secrete a variety of factors, called lymphokines, and they may induce, augment or even switch off immune responses.

CDD—Were T cells shown to play a role in cancer or cancer development?

Lymphocytes had for long been noticed in histological sections of tumours, and at the time of my early work, it was believed by some scientists that they might be involved in restraining tumour growth. In 1963, I applied 3,4-benzopyrene to young adult mice that had been neonatally thymectomized or sham-operated. Papillomas occurred in both sets of mice but reached a larger area in the NTx mice. Most importantly, by 180 days 12% of skin tumours in the NTx mice became malignant in contrast to only 4% in the control mice. My words in the conclusion of my 1963 Nature paper on this topic are: “Interference with the cellular immune mechanism may be necessary, in some cases, to allow the full expression of a carcinogenic process” (Fig. 3).

**Fig. 3:**
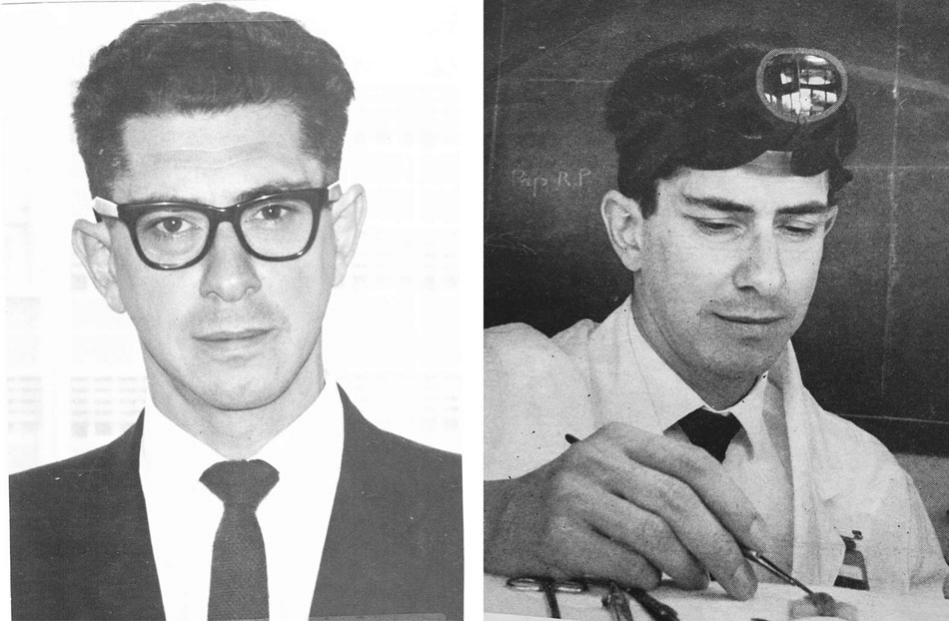
Left, Jacques in 1966. Right, Jacques performing neonatal thymectomy in 1967.

CDD—Does apoptosis occur during thymus development?

Yes, the proapoptotic Bcl-2 family member Bim plays a critical role in T-cell development. Andreas Strasser and his group showed that it is essential for the intra-thymic deletion of T cells that are autoreactive. Bim is also involved in some immune reactions that delete peripheral T cells.

CDD—What are the clinical implications of your work?

The thymus, once believed to be a useless vestigial organ populated with cells—which, in 1963, were considered by Nobel Laureate Sir Peter Medawar “as an evolutionary accident of no very great significance”—is producing T cells that play a role in the entire spectrum of tissue physiology and pathology. They are involved in inflammation, in infections, in vaccination, in allergies, in immunodeficiency, in autoimmunity, in rejection of tissue and organ transplants, in dysbiosis, and in reactions not usually considered to be immunological, e.g. in pregnancy, in metabolism, and in tissue repair. It is very exciting for me to see that T cells can now be used with spectacular success in immunotherapy of some cancers and may no doubt eventually replace radiotherapy and chemotherapy in the treatment of many tumours.

CDD: what advice would you give young scientists?

To young scientists I would say the following.

“You, young people, you are the future of your country. You are also in many ways the future of the world! That is because you have chosen a career in science. And it is only through science that humanity can improve its lot on many fronts. It is by scientific knowledge that we can increase the world’s food supply and eventually end poverty. It is through scientific knowledge that we can reverse the effects of climate change that threaten our planet and all the species that live there. It is by the use of scientific knowledge that we can change our energy production methods to those that use renewable and clean energy. It is through scientific knowledge that we can prevent epidemics and we can ameliorate or even cure many diseases, now including cancer.

Science is a worthwhile activity. It is something you can enjoy doing (while being paid for it). Of course, you need to work hard. You need to ask the right questions and to formulate a hypothesis that can be tested experimentally. And then you need patience, persistence, and perseverance. You need to repeat your experiments more than once and you need to thoroughly check your data. And if your data are solid, you need not be disturbed if your interpretation is not widely accepted. You may be wrong in your interpretation but that does not matter, as long as your data are firm and reproducible. And you need not to be discouraged if your work cannot be translated into something useful that can for example be used in a clinical context. It can take many years from bench to bedside. In my case, it took nearly 60 years from the discovery of thymus function and of T and B cells to the use of T and B cells as cells that produce monoclonal antibody, and as killer cells in cancer immunotherapy.

In conclusion I would just say that the future belongs to you. Hence good luck, work hard and have fun whilst doing so”.

CDD: would you provide some early readings for young scientists?

Certainly, here are some:

Baxter AG. Germ Warfare. 2000. Sydney: Allen & Unwin.

Davey GM, Kurts C, Miller JF, Bouillet P, Strasser A, Brooks AG, et al. Peripheral deletion of autoreactive CD8 T cells by cross presentation of self-antigen occurs by a Bcl-2-inhibitable pathway mediated by Bim. J Exp Med. 2002;196:947–55.

De Burgh PM, Miller JF. Cellular control in virus infection. Nature. 1955;175(4456):550.

Hedrick SM, Cohen DI, Nielsen EA, Davis MM. Isolation of cDNA clones encoding T cell-specific membrane-associated proteins. Nature. 1984;308:149–53.

Medawar PB. Induction and intuition in scientific thought. 1969. London: Methuen.

Medawar PB. The Art of the Soluble. 1968. London: Methuen.

Miller JF, Grant GA, Roe FJ. Effect of thymectomy on the induction of skin tumours by 3,4-benzopyrene. Nature. 1963;199:920–2.

Miller JF, Mitchell GF, Weiss NS. Cellular basis of the immunological defects in thymectomized mice. Nature. 1967;214:992–7.

Miller JF, Mitchell GF. Cell to cell interaction in the immune response. I. Hemolysin-forming cells in neonatally thymectomized mice reconstituted with thymus or thoracic duct lymphocytes. J Exp Med. 1968;128:801–20.

Miller JF. Analysis of the thymus influence in leukaemogenesis. Nature. 1961;191:248–9.

Miller JF. Effect of neonatal thymectomy on the immunological responsiveness of the mouse. Proc R Soc. 1962: 156B:415–28.

Miller JF. Fate of subcutaneous thymus grafts in thymectomized mice inoculated with leukaemic filtrate. Nature. 1959;184(Suppl 23):1809–10.

Miller JF. Immunological function of the thymus. Lancet. 1961;2:748–9.

Miller JF. Immunological significance of the thymus of the adult mouse. Nature. 1962;195:1318–9.

Miller JF. Recovery of leukaemogenic agent from nonleukaemic tissues of thymectomized mice. Nature. 1960;187:703.

Miller JF. Role of the thymus in murine leukaemia. Nature. 1959;183:1069.

Miller JF. Studies on mouse leukaemia. The fate of thymus homografts in immunologically tolerant mice. Br J Cancer. 1960;14:244–55.

Popper KR. The growth of Scientific Knowledge. 1962. London: Routledge.

Richtel M. An elegant defence. 2019. New York: Harper Collins.

Sheffield M. Critical paths to achievement. 2001. Sydney: Capital Technic Group.

Siu G, Kronenberg M, Strauss E, Haars R, Mak TW, Hood L. The structure, rearrangement and expression of D beta gene segments of the murine T-cell antigen receptor. Nature. 1984;311(5984):344–50.

Yanagi Y, Yoshikai Y, Leggett K, Clark SP, Aleksander I, Mak TW. A human T cell-specific cDNA clone encodes a protein having extensive homology to immunoglobulin chains. Nature. 1984;308:145–9.

Yoshikai Y, Anatoniou D, Clark SP, Yanagi Y, Sangster R, Van den Elsen P, Terhorst C, Mak TW. Sequence and expression of transcripts of the human T-cell receptor beta-chain genes. Nature. 1984;312(5994):521–4.

